# Systolic blood pressure targets below 120 mm Hg are associated with reduced mortality: A meta‐analysis

**DOI:** 10.1111/joim.20078

**Published:** 2025-03-05

**Authors:** Felix Bergmann, Marlene Prager, Lena Pracher, Rebecca Sawodny, Gloria M. Steiner‐Gager, Bernhard Richter, Bernd Jilma, Markus Zeitlinger, Georg Gelbenegger, Anselm Jorda

**Affiliations:** ^1^ Department of Clinical Pharmacology Medical University of Vienna Vienna Austria; ^2^ Department of Pathology Medical University of Vienna Vienna Austria; ^3^ Department of Medicine II Division of Cardiology Medical University of Vienna Vienna Austria

**Keywords:** all‐cause mortality, antihypertensives, heart failure, Hypertension, myocardial infarction, randomized, stroke

## Abstract

**Background:**

The optimal systolic blood pressure (SBP) target in patients with increased cardiovascular risk remains uncertain. This study evaluated the efficacy and safety of intensive SBP control (<120 mm Hg) compared to standard SBP control (<140 mm Hg) in patients with increased cardiovascular risk.

**Methods:**

We conducted a systematic search of PubMed, Embase, Web of Science, and Cochrane Library for RCTs published from database inception through November 2024 that compared intensive SBP control (<120 mm Hg) with standard SBP control (<140 mm Hg) in adults with high cardiovascular risk. Efficacy outcomes included all‐cause mortality, major adverse cardiovascular events (MACE), cardiovascular death, stroke, myocardial infarction (MI), and heart failure. Safety outcomes included hypotension, syncope, arrhythmia, acute kidney injury, and electrolyte abnormalities.

**Results:**

Five RCTs comprising 39,434 patients were included. The all‐cause mortality was significantly lower in the intensive SBP control group (672 of 19,712 [3.4%]) compared to the standard SBP control group (778 of 19,722 [3.9%]) (risk ratio 0.87 [95% confidence interval, 0.76–0.99, *p* = 0.03]). The incidence of MACE, cardiovascular death, MI, stroke, and heart failure was significantly lower in the intensive SBP control group as compared to standard SBP control group. The treatment effect (MACE) was consistent across all subgroups. Conversely, intensive SBP control was associated with an increased risk of hypotension, syncope, arrhythmia, acute kidney injury, and electrolyte abnormalities.

**Conclusions:**

Targeting intensive SBP control to less than 120 mm Hg was associated with a lower incidence of all‐cause mortality and MACE but a higher incidence of adverse events.

## Graphical abstract

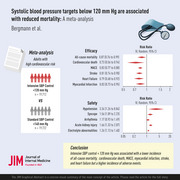

## Introduction

Elevated blood pressure (BP) is regarded as a major risk factor for cardiovascular disease (CVD) and significantly contributes to morbidity and mortality worldwide [1, 2]. A comprehensive meta‐analysis determined that lowering systolic BP (SBP) by 5 mm Hg, regardless of baseline BP, may decrease the risk of major cardiovascular events by approximately 10% [3]. The recently published 2024 European Society of Hypertension guidelines recommend initially lowering SBP to below 140 mm Hg and diastolic BP (DBP) to below 80 mm Hg [4]. For most patients under 80 years of age, the SBP target is below 130 mm Hg [4]. This recommendation is in line with the recommendations of the American College of Cardiology/American Heart Association [5], which also suggest targets of 130/80 mm Hg, especially in patients with increased cardiovascular risk. Although the benefits of reducing BP further to below 120/70 mm Hg remain uncertain, recent evidence suggests that more intensive BP control may offer additional benefits for patients with increased cardiovascular risk [6].

Large trials investigating the potential benefit of lowering SBP to more intensive targets of less than 120 mm Hg have yielded conflicting results. The SPRINT trial [7] was the first to show that targeting an SBP below 120 mm Hg significantly reduced the incidence of major adverse cardiovascular events (MACEs) and all‐cause mortality compared to a target of less than 140 mm Hg in patients with increased cardiovascular risk. In contrast, the ACCORD trial [8], which examined this approach in patients with diabetes, failed to demonstrate a significant reduction in MACE. Furthermore, excessively low BP has also been linked to increased rates of cardiovascular events and other adverse outcomes, including hypotension and syncope [9]. Consequently, the optimal BP target remains debated.

Considering recent large randomized controlled trials, we conducted a meta‐analysis evaluating the efficacy and safety of intensive BP control to an SBP lower than 120 mm Hg versus standard BP control to lower than 140 mm Hg in patients with increased cardiovascular risk.

## Methods

### Study registration

This meta‐analysis was conducted according to the Cochrane Handbook [10], was reported following the Preferred Reporting Items for Systematic Reviews and Meta‐Analyses (PRISMA) [11] guidelines, and was registered at PROSPERO with the identifier CRD42024575910. The present study did not require approval by the ethics committee.

### Data sources and study selection

We conducted a systematic literature search and meta‐analysis of randomized controlled trials to evaluate the summary effect estimate of intensive BP control (SBP target <120 mm Hg) versus standard BP control (SBP target <140 mm Hg) on all‐cause mortality in patients aged 18 years or older. We searched major online databases, including PubMed, Web of Science, Embase, and Cochrane Library, for peer‐reviewed, randomized controlled clinical trials published in English up to November 28, 2024. The core search terms used were as follows: (blood pressure OR hypertension) and (cardiovascular risk OR myocardial infarction OR stroke OR heart failure) and (120 mm Hg OR intensive blood pressure), along with relevant Medical Subject Headings terms or synonyms. Table S1 contains the complete search input for each database. Furthermore, we screened prior systematic reviews and the references of included studies to identify further relevant studies.

Two reviewers (L.P. and F.B.) independently screened studies based on their titles, abstracts, and full texts to identify those that met the inclusion criteria. If there were discrepancies in selection, a third reviewer (A.J.) was consulted to reach a consensus. The key inclusion criteria were as follows: (i) a randomized controlled clinical trial design, (ii) comparison of intensive BP control (SBP target <120 mm Hg) versus standard BP control (SBP target <140 mm Hg), (iii) study population consisted of patients with high cardiovascular risk, (iv) reporting all‐cause mortality, and (v) patients 18 years or older. Exclusion criteria were non‐randomized trials, retrospective analyses, abstracts, case‐reports, observational data, reviews, trial protocols, and trials that repeatedly published data from the same data set.

### Data extraction and outcomes

This meta‐analysis analyzed all outcomes following the intention‐to‐treat principle from the individual studies. The present analysis only included observed data from the respective trials. We performed no imputation. The handling of missing data in the individual trials is described in the respective publications.

The primary outcome specified for this meta‐analysis was all‐cause mortality during the whole observational period of the respective studies. Secondary outcomes included MACE (the respective definitions of MACE for each included study can be found in Table S2), cardiovascular death, stroke, MI, and heart failure. Safety outcomes comprised the occurrence of hypotension, syncope, and arrhythmia (as defined by bradycardia or arrhythmia [8], tachyarrhythmia [12], or bradycardia [7]). Safety outcomes refer solely to the documented serious adverse events, which are defined as events that are life‐threatening, result in permanent disability, or require hospitalization. Although MACE was the primary endpoint in the individual trials, we selected all‐cause mortality as the primary endpoint for three key reasons. First, MACE was chosen in the original trials because it provides greater statistical power, which is of less importance in this large pooled analysis. Second, although MACE addresses critical outcomes of suboptimal antihypertensive treatment, it may not fully capture the broader impact on health outcomes. Third, inconsistencies in the definition of MACE across studies could introduce bias into the pooled effect estimates.

Importantly, the individual trials reported their subgroup analyses for the respective primary outcomes, which in most cases was the occurrence of MACE. Subsequently, we were only able to pool these published subgroup analyses. These analyses considered sex, elderly versus non‐elderly patients (respective definitions provided in Table S3), patients with diabetes versus those without, patients with prior CVD versus those without, and mean baseline SBP ≤145 versus >145 mm Hg.

Two authors (F.B. and A.J.) assessed the overall certainty of evidence for the primary outcome using the Grading of Recommendations, Assessment, Development, and Evaluations [13] guidelines. The Cochrane risk of bias (RoB) tool was used to evaluate the RoB in the included trials.

### Data synthesis and statistical analysis

Categorical variables are presented as numbers with percentages (%). Continuous variables are summarized as means with standard deviations or medians with interquartile ranges, depending on the data distribution. We computed pooled risk ratios (RRs) using a random‐effects model to address the variability among studies. This model was selected due to the methodological diversity of the studies included. We present RRs along with 95% confidence intervals (95% CI) and *p*‐values for the summary estimates. For the primary outcome, we additionally calculated the risk difference (RD) and number needed to treat (NNT) using the inverse‐variance method within a random‐effects framework.

Heterogeneity across studies was evaluated using Chi‐square statistics and the Higgins *I* ^2^ statistic. *I* ^2^ values were categorized as minimal (0%–25%), moderate (26%–50%), high (51%–75%), and very high (76%–100%) inconsistency [14]. Differences between subgroups were examined through tests for heterogeneity within subgroups. Sensitivity analyses were conducted by sequentially excluding each individual trial and by comparing results from a fixed‐effects model to those from a random‐effects model. The Mantel–Haenszel method was utilized within the fixed‐effects framework.

We report unadjusted *p*‐values, with statistical significance defined as a two‐sided alpha of 0.05. Statistical analyses were performed using Review Manager (version 5.4.1, 2014, Copenhagen: The Nordic Cochrane Centre, The Cochrane Collaboration) and R (R version 2024.04.2+764).

## Results

**Fig. 1:**
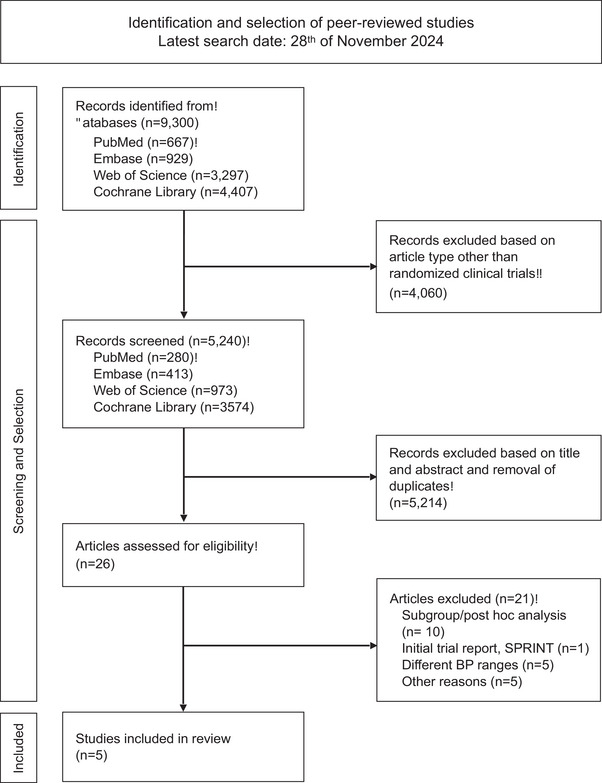
Preferred Reporting Items for Systematic Reviews and Meta‐Analyses (PRISMA) chart of study identification and selection.

The literature search yielded a total of 9300 citations. After excluding non‐randomized trials, 5240 citations remained. Of these, 5214 were excluded based on titles, abstracts, and duplicate removal. We then reviewed the full‐text articles of the remaining 26 citations, of which five were included in the final analysis. The PRISMA flow chart detailing the selection process is shown in Fig. 1.

### Study characteristics

Our final analysis included 5 trials with a total of 39,434 patients [7, 8, 12, 15, 16]. Table 1 summarizes the key characteristics of these studies, whereas extended details are available in Table S4. The ACCORD trial [8] took place in the United States and Canada; the BPROAD and ESPRIT trials [12, 16] were conducted in China; the RESPECT study [15] was carried out in Japan; and the SPRINT trial [7] included patients from the United States [12]. Sample sizes ranged from 1263 to 12,821 patients. The definitions of a high cardiovascular risk differed between the trials but included diabetes, history of stroke, advanced chronic kidney disease, age, dyslipidemia, and others. The complete list of inclusion criteria can be found in Table S4. The study populations were patients with Type 2 diabetes mellitus (ACCORD and BPROAD trials) [8, 16], patients with a history of stroke (RESPECT trial) [15], patients at increased risk for CVD without diabetes or previous stroke (SPRINT trial) [7], and patients at increased risk for CVD with or without diabetes or previous stroke (ESPRIT trial) [12]. The primary outcome was MACE in the ACCORD [8], BPROAD [16], SPRINT [7], and ESPRIT [12] trials and recurrent stroke in the RESPECT [15] study. All studies included data on all‐cause mortality as a secondary outcome. An overview of patient characteristics in the included studies is detailed in Table S5. The follow‐up period between studies was similar, ranging from 3.3 to 4.7 years (Table 1).

**Table 1:** Key study characteristics of the included trials.

Study	Year	Geographical region	Number of sites	Study population	Total sample size	Pimary outcome	Follow‐up^a^
ACCORD	2010	USA, Canada	77	Type 2 diabetes mellitus	4733	MACE	4.7 years^b^
BPROAD	2024	China	145	Type 2 diabetes mellitus	12,821	MACE	4.2 years
ESPRIT	2024	China	116	High cardiovascular risk, with and without diabetes or previous stroke	11,255	MACE	3.4 (3.0–3.4) years
RESPECT	2019	Japan	140	History of stroke within previous 3 years	1263	Recurrent stroke	3.9 ± 1.5 years
SPRINT	2021	United States	102	Increased risk for cardiovascular disease but no diabetes or previous stroke	9361	MACE	3.33 years^c^

*Note*: Data are presented as mean ± SD or median (IQR) where applicable.

Abbreviation: MACE, major adverse cardiovascular events.

^a^ Refers to the follow up period of the respective study's primary outcome.

^b^ Data presented as mean.

^c^ Data presented as median.

### Primary outcome

**Fig. 2:**
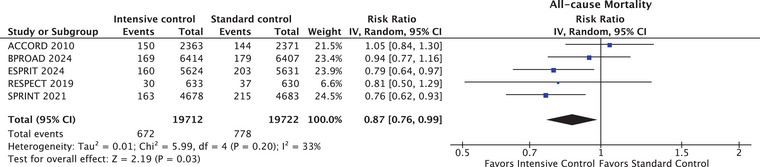
Forest plot depicting the association between intensive versus standard blood pressure control and all‐cause mortality.

Table 2 presents the efficacy and safety outcomes of this meta‐analysis. All‐cause mortality was reported by all studies (39,434 patients). The all‐cause mortality was significantly lower in the intensive BP control group (672 of 19,712 patients [3.4%]) compared to the standard BP control group (778 of 19,722 patients [3.9%]), resulting in a percentage RR of 0.87 (95% CI, 0.76–0.99 *p* = 0.03; *I* ^2^ = 33%) (Fig. 2). The corresponding percentage absolute RD was −0.5% (95% CI, −1.03% to −0.06%) (RD, −0.005 [95% CI, −0.010 to −0.001)]; *p* = 0.028; *I* ^2^ = 34%). The estimated NNT to prevent one death was 185 (95% CI, 97–1667). The certainty of evidence of the primary endpoint was considered low and is detailed in Table S6.

**Table 2:** Efficacy and safety outcomes

	Studies	Intensive BP control group, *n* (%)	Standard BP control group, *n* (%)	Risk ratio (95% CI)
**Efficacy outcomes**				
All‐cause mortality	[7, 8, 12, 15, 16]	672/19,712 (3.4)	778/19,722 (3.9)	0.87 (0.76–0.99)
MACE	[7, 8, 12, 15, 16]	1458/19,712 (7.4)	1765/19,722 (8.9)	0.83 (0.77–0.88)
Cardiovascular death	[7, 8, 12, 16]	220/19,079 (1.2)	305/19,092 (1.6)	0.73 (0.56–0.94)
Stroke	[7, 8, 12, 15, 16]	690/19,712 (3.5)	851/19,722 (4.3)	0.81 (0.74–0.90)
Myocardial infarction	[7, 8, 12, 15, 16]	383/19,712 (1.9)	462/19,722 (2.3)	0.83 (0.73–0.95)
Heart failure	[7, 8, 12, 16]	305/19,079 (1.6)	386/19,092 (2.0)	0.79 (0.68–0.92)
**Safety outcomes**				
Hypotension	[7, 8, 12, 16]	131/19,078 (0.7)	63/19,092 (0.3)	3.34 (1.26–8.87)
Syncope	[7, 8, 12, 15, 16]	149/19,711 (0.8)	100/19,722 (0.5)	1.56 (1.09–2.23)
Arrhythmia	[7, 8, 12, 16]	246/19,078 (1.3)	211/19,092 (1.1)	1.17 (0.93–1.49)
Acute kidney injury	[7, 8, 12, 16]	205/19,078 (1.1)	123/19,092 (0.6)	1.66 (1.33–2.07)
Electrolyte abnormalities	[7, 8, 12, 16]	1,204/19,078 (6.3)	950/19,092 (5.0)	1.26 (1.13–1.40)

Abbreviations: 95% CI, 95% confidence interval; BP, blood pressure; MACE, major adverse cardiovascular events.

### Secondary outcomes

#### MACE

**Fig. 3:**
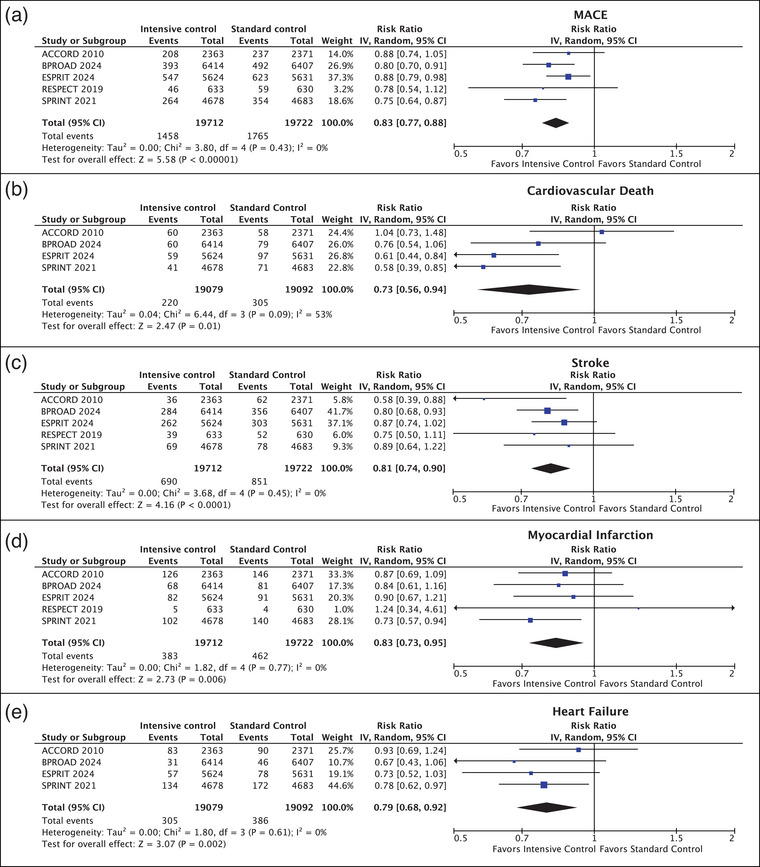
Forest plot depicting the efficacy outcomes of intensive versus standard blood pressure control: (a) incidence of major adverse cardiovascular events (MACE), (b) cardiovascular death, (c) incidence of stroke, (d) incidence of myocardial infarction, and (e) incidence of heart failure.

All studies (39,434 patients) reported the incidence of MACE. Intensive BP control was associated with a statistically significant reduction in the risk of developing MACE compared to those in the standard control group. MACE occurred in 1458 of 19,712 (7.4%) of patients in the intensive BP control group and in 1765 of 19,722 (8.9%) of patients in the standard control group (RR 0.83 [95% CI, 0.77–0.88)]; *p* < 0.001; *I* ^2^ = 0%) (Fig. 3a).

#### Cardiovascular death

Four studies (38,171 patients) reported the incidence of cardiovascular death. The incidence of cardiovascular death was significantly lower in the intensive BP control group (220 of 19,079 patients [1.2%]) compared to the standard control group (305 of 19,092 patients [1.6%]) (RR 0.73 [95% CI, 0.56–10.94]; *p* = 0.01; *I* ^2^ = 53%) (Fig. 3b).

#### Stroke

All studies (39,434 patients) reported the incidence of stroke. Intensive BP control resulted in a statistically significant reduction in the risk of developing stroke compared to those in the standard control group. Stroke occurred in 690 of 19,712 (3.5%) of patients in the intensive BP control group and in 851 of 19,092 (4.3%) of patients in the standard control group (RR 0.81 [95% CI, 0.74–0.90]; *p* < 0.001; *I* ^2^ = 0%) (Fig. 3c).

#### Myocardial infarction

All studies (39,434 patients) reported the incidence of MI. Intensive BP control was associated with a lower risk of developing an MI compared to those in the standard control group. MI occurred in 383 of 19,712 (1.9%) of patients in the intensive BP control group and in 462 of 19,722 (2.3%) of patients in the standard control group (RR, 0.83 [95% CI, 0.73–0.95]; *p* = 0.006; *I* ^2^ = 0%) (Fig. 3d).

#### Heart failure

Four studies (38,171 patients) reported data on the incidence of heart failure. The incidence of heart failure was significantly lower in the intensive BP control group (305 of 19,712 patients [1.9%]) compared to the standard control group (386 of 19,722 patients [2.3%]) (RR 0.83 [95% CI, 0.73–0.95]; *p* = 0.002; *I* ^2^ = 0%) (Fig. 3e).

### Adverse events

**Fig. 4:**
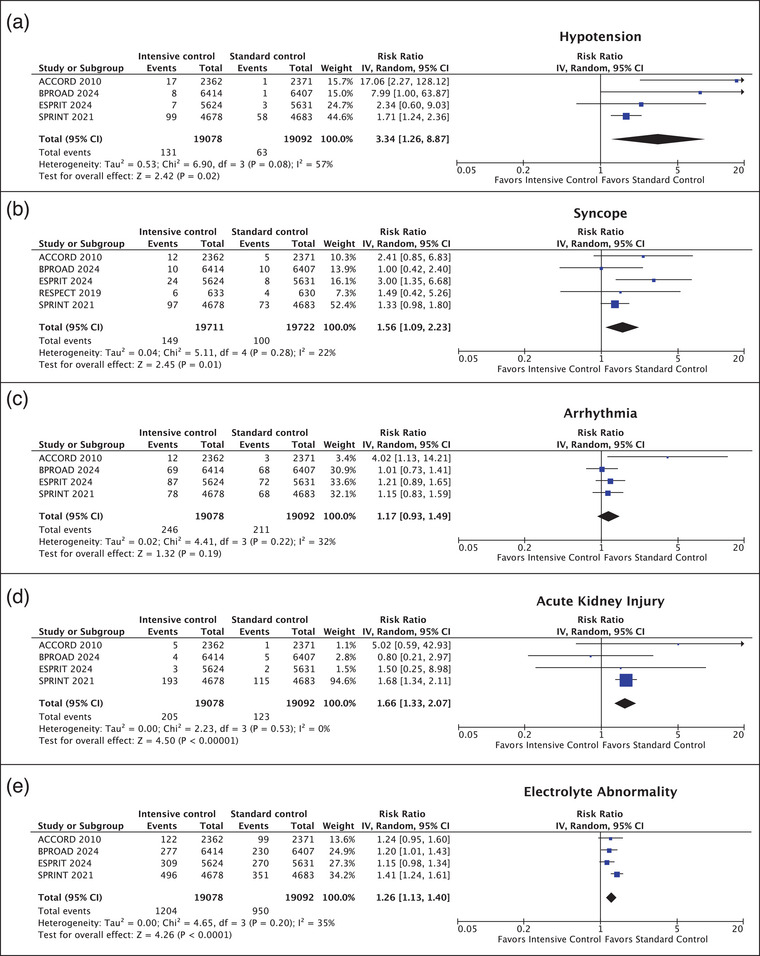
Forrest plot depicting the safety outcomes of intensive versus standard blood pressure control: (a) hypotension, (b) syncope, (c) arrhythmia, (d) acute kidney injury, (e) electrolyte abnormalities. Electrolyte abnormalities were defined by a serum sodium of <130 mmol/L, a serum sodium of >150 mmol/L, a serum potassium of <3 mmol/L (SPRINT, BPROAD, and ESPRIT) or <3.2 mmol/L (ACCORD), and a serum potassium of >5.5 mmol/L (SPRINT, BPROAD, and ESPRIT) or >5.9 mmol/L (ACCORD).

Fig. 4 presents an overview of the safety analysis. Four studies (38,170 patients) reported the incidence of hypotension. Intensive BP control was associated with an increased risk of developing hypotension compared to those in the standard control group. Hypotension occurred in 131 of 19,078 (0.7%) of patients in the intensive BP control group and in 63 of 19,092 (0.3%) of patients in the standard control group (RR, 3.34 [95% CI, 1.26–8.87]; *p* = 0.02; *I* ^2^ = 57%) (Fig. 4a). All studies (39,433 patients) reported the occurrence of syncope, which was significantly higher in the intensive BP control group (149 of 19,711 patients [0.8%]) compared to the standard control group (100 of 19,722 patients [0.5%]) (RR 1.56 [95% CI, 1.09–2.23]; *p* = 0.01; *I* ^2^ = 22%) (Fig. 4b). Four studies (38,170 patients) reported the incidence of arrhythmia. Intensive BP control was not associated with an increased risk of developing arrhythmia compared to those in the standard control group. Arrhythmia occurred in 246 of 19,078 (1.3%) of patients in the intensive BP control group and in 211 of 19,092 (1.1%) of patients in the standard control group (RR, 1.17 [95% CI, 0.93–1.49]; *p* = 0.19; *I* ^2^ = 32%) (Fig. 4c).

Acute kidney injury occurred more frequently in the intensive control group (205 of 19,078 [1.1%]) than in the standard BP control group (123 of 19,092 [0.6%]) (RR, 1.66 [95% CI, 1.33–2.07]; *p* < 0.001; *I* ^2^ = 0%) (Fig. 4d). Electrolyte abnormalities occurred more frequently in the intensive control group (1204 of 19,078 [6.3%]) than in the standard BP control group (950 of 19,092 [5.0%]) (RR, 1.26 [95% CI, 1.13–1.40]; *p* < 0.001; *I* ^2^ = 35%) (Fig. 4e).

### Subgroup analyses

No significant subgroup differences between sexes were found in the association of intensive versus standard BP control with the incidence of MACE (Fig. S1). The incidence of MACE was lower in the intensive BP control group than in the standard control group in both male (RR, 0.82 [95% CI, 0.74–0.90]) and female patients (RR, 0.85 [95% CI, 0.76–0.95]). No significant differences between elderly and non‐elderly patients were found in the impact of BP control on MACE incidence (Fig. S2). The incidence of MACE was lower in the intensive BP control group in elderly patients (RR, 0.83 [95% CI, 0.74–0.92]) and in non‐elderly patients (RR, 0.85 [95% CI, 0.78–0.94]) (test for subgroup differences; Chi^2^ = 0.23; *p* = 0.63). Similarly, no significant differences were found between patients with versus without diabetes (Fig. S3). The incidence of MACE was lower in the intensive BP control group in patients with diabetes (RR, 0.85 [95% CI, 0.78–0.92]) and in patients without diabetes (RR, 0.79 [95% CI, 0.69–0.92]) (test for subgroup differences; Chi^2^ = 0.63; *p* = 0.43). No significant differences were found between patients with previous CVD or without previous CVD (Fig. S4). There was an association between the incidence of MACE and BP group in patients with previous CVD (RR, 0.87 [95% CI, 0.78–0.96]) and patients without previous CVD (RR, 0.80 [95% CI, 0.71–0.89]) (test for subgroup differences; Chi^2^ = 1.21; *p* = 0.27). Finally, there was an association between the incidence of MACE and BP group in patients with a mean baseline SBP of ≤145 mm Hg (RR, 0.85 [95% CI, 0.77–0.94]) and patients with a mean baseline SBP of >145 mm Hg (RR, 0.83 [95% CI, 0.75–0.91]) (test for subgroup differences; Chi^2^ = 0.22; *p* < 0.001) (Fig. S5).

### Sensitivity analyses

Switching from a random‐effects model to a fixed‐effects model had no substantial impact on the primary outcome of this analysis (RR, 0.87 [95% CI, 0.78–0.96]; *p* = 0.005; *I* ^2^ = 33%). Sequential omission of each study revealed a change of significance of the primary outcome when omitting the ESPRIT trial (RR, 0.89 [95% CI, 0.76–1.04]; *p* = 0.16; *I* ^2^ = 39%), the RESPECT trial (RR, 0.87 [95% CI, 0.75–1.01]; *p* = 0.07; *I* ^2^ = 49%), and the SPRINT trial (RR, 0.91 [95% CI, 0.79–1.04]; *p* = 0.15; *I* ^2^ = 20%).

### Risk of bias and certainty of evidence

The most relevant RoB arose from the open‐label study design of the included trials, which could potentially impact the lifestyle of patients and the level of care of treating physicians (e.g., more visits to reach the more intensive target) (Table S6). Theoretically, adjudication could have been blinded to minimize the risk of detection bias, but this was not feasible in any of the trials. Overall, we rate the certainty of the evidence as low, mainly due to the open‐label design, the small absolute effect size, and the inconsistency in the assessment of BP in the SPRINT trial (Table S7).

## Discussion

Elevated BP significantly increases CVD risk, yet optimal targets remain debated due to inconsistent outcomes and concerns about side effects [17, 18]. The recent update of the ESC hypertension guidelines from August 2024 considered only three of the five studies incorporated in the present meta‐analysis. Compared with the previous version, which recommended targeting SBP values of 140/90 mm Hg for all patients, with a further target of 130/80 mm Hg or lower if tolerated, the revised guideline now suggests targeting SBP values of 120–129 mm Hg, provided the treatment is well tolerated [19].

The present study evaluated 5 large, randomized clinical trials and incorporated 39,434 patients. To the best of our knowledge, this meta‐analysis was the first to indicate that targeting an SBP of <120 mm Hg, compared to less than 140 mm Hg, was associated with a reduced risk of all‐cause mortality, cardiovascular death, and MACE. However, this cardiovascular benefit was accompanied by an increased rate of serious adverse events, including syncope and arrhythmia, due to the lower BP levels achieved.

Although several large RCTs have aimed to identify ideal BP targets, the optimal thresholds for patients with increased cardiovascular risk continue to be a subject of significant debate due to inconsistent outcomes [18]. Both the ACCORD and BPROAD studies only included patients with Type 2 diabetes [8, 16]. Although the ACCORD trial failed to demonstrate a significant reduction in the incidence of MACE, the BPROAD study was able to demonstrate a benefit of intensive SBP control. A recent meta‐analysis of individual participant‐level data in patients with and without diabetes found that lowering SBP reduced the risk of major cardiovascular events in both groups, with a weaker relative risk reduction in participants with Type 2 diabetes compared to those without, although absolute risk reductions were similar between the two groups [20]. Furthermore, another large meta‐analysis, which included over 70,000 patients, demonstrated that antihypertensive treatment reduced mortality and cardiovascular risk in people with diabetes and SBP above 140 mm Hg but increased cardiovascular death risk when blood pressure is below 140 mm Hg [21]. In contrast, our subgroup analysis found no significant differences between patients with and without diabetes, suggesting that intensive SBP with targets lower than 120 mm Hg control may offer potential benefits for both groups. The SPRINT trial was the first to show that intensive BP control significantly reduced rates of MACE and all‐cause mortality in patients with increased cardiovascular risk [7]. However, there have been concerns regarding the validity and generalizability of the study's results, because the trial excluded patients with Type 2 diabetes and a history of stroke [22]. Furthermore, the SPRINT trial employed an unattended BP measurement technique, which may have resulted in lower BP values compared to those obtained in other trials, as demonstrated by Seo et al. [23, 24]. As argued by Kjeldsen et al., the measurements obtained in the SPRINT trial may not be directly comparable to the BPs in the other trials [23]. Notably, the omission of SPRINT trial from the pooled analysis rendered the association statistically non‐significant, which poses an important limitation of this study. The RESPECT trial investigated the effect of intensive BP treatment in patients with a history of stroke but failed to demonstrate a significant reduction in the primary outcome of incidence of recurrent stroke or the secondary outcome of all‐cause mortality [15]. Similar to the ACCORD trial, the RESPECT study did not achieve sufficient power to draw statistical significance for the primary outcome. However, the authors conducted a pooled meta‐analysis that included three additional trials and found a significant benefit of intensive BP lowering compared with standard treatment for stroke recurrence [25, 26, 27]. The ESPRIT trial performed a subgroup analysis comparing patients with and without prior strokes and found no significant heterogeneity between the groups concerning their primary outcome of MACE [12]. Consistent with the results of the SPRINT trial, the ESPRIT study found a significant reduction in the incidence of MACE and all‐cause mortality in the intensive BP control group in patients with high cardiovascular risk with and without diabetes or previous stroke [12]. Actual BPs achieved in the intensive BP control groups differed among the studies, ranging from 119 mm Hg in the ESPRIT and ACCORD trials to 127 mm Hg in the RESPECT trial. Although achieving the BP target likely interferes with the treatment effect, our analysis used the intention‐to‐treat data irrespective of target attainment.

Importantly, the reduced risk of all‐cause mortality, cardiovascular death, and incidence of MACE and stroke was accompanied by a higher risk of developing severe adverse events such as arrhythmia and syncope. Furthermore, the SPRINT and ESPRIT trials, along with other secondary analyses, have noted that intensive BP control may increase the risk of sustained renal function decline, although only a few participants developed end‐stage renal disease [7, 12, 28, 29]. Therefore, clinicians should balance the potential benefits of lowering SBP below current recommendations with the associated risks and aim to maintain the lowest tolerable SBP level for each patient.

This meta‐analysis [32] extends the findings of previous landmark trials, such as the PROGRESS [30] and HOPE [31] trials, which showed the beneficial effects of antihypertensive treatment with ACE‐inhibitors in patients with or at high risk for CVD, even in the absence of hypertension. Although the ideal choice of antihypertensive drugs remains debated, the ACCOMPLISH trial [32], which also included high‐risk patients, demonstrated the superiority of ACE‐inhibitors combined with calcium‐channel blockers compared with ACE‐inhibitors combined with HCT, independent of the reduction of SBP. Therefore, antihypertensive treatment may offer additional benefits beyond solely reducing BP.

We acknowledge several limitations of this meta‐analysis. First, the trial‐level design has inherent limitations, as previously discussed [33]. Second, the trials included in this meta‐analysis involved patients with varying levels of cardiovascular risk. Third, despite the variation in observational periods among the included studies, the trials lacked sufficient data granularity to adjust for these differences. Furthermore, considering the proportional hazards assumption, RRs were assumed to stay constant over time. Fourth, although two studies reported the achieved DBP, no analyses were conducted to investigate the effect of lowering DBP on the outcomes. Fifth, there was a moderate inconsistency for the endpoint stroke. Because the ESPRIT and BPROAD trials took place in China and the RESPECT trial was carried out in Japan, this inconsistency may be explained by the higher incidence of stroke in East‐Asian populations compared to Western countries [34]. Hence, the generalizability of these findings to Western populations remains uncertain, and further validation studies are necessary before lower SBP targets can be broadly adopted in clinical practice outside East Asia.

Furthermore, although our analysis compared lowering SBP to lower than 140 versus to lower than 120, no comparison between SBP targets of lower than 130 versus lower than 120 is currently available, which warrants further investigation. Finally, the trials included in this analysis used varying definitions for some outcomes, such as MACE and arrhythmia, and differed in the categorization of certain subgroups, such as age.

## Conclusion

In patients with increased cardiovascular risk, an intensive BP target below 120 mm Hg compared with a standard BP target below 140 mm Hg was associated with a reduction in all‐cause mortality, cardiovascular death, and a reduced incidence of MACE, MI, stroke, and heart failure. There was a significant increase in the incidence of hypotension, syncope, acute kidney injury, and electrolyte abnormalities in the intensive control group.

## Author contributions

All listed authors meet all four criteria for authorship in the ICMJE Recommendations. Felix Bergmann, Georg Gelbenegger, and Anselm Jorda conceived the study idea. Felix Bergmann, Marlene Prager, and Lena Pracher performed the research and extracted the data. Felix Bergmann and Anselm Jorda performed the statistical analysis and prepared the figures and tables. Felix Bergmann drafted the manuscript. All authors critically revised the manuscript and approved the final version of the manuscript.

## Conflict of interest statement

The authors declare no conflicts of interest.

## Funding information

Internal funding.

## Supporting information




**Table S1**: Precise search strategy and number of records found in each database (Search conducted on November 28, 2024).
**Table S2**: Definition of the outcome MACE of included studies.
**Table S3**: Definition of elderly versus non‐elderly for subgroup analysis.
**Table S4**: Extended trial characteristics.
**Table S5**: Baseline characteristics of the study populations in the included trials.
**Table S6**: Assessment of level of certainty of evidence according to GRADE recommendations.
**Table S7**: Risk of Bias Assessment according to the Revised Cochrane risk‐of‐bias tool for randomized trials (RoB 2).
**Figure S1**: Forrest plot depicting the subgroup comparison of the effect of intensive blood pressure control on the incidence of MACE in male versus female patients.
**Figure S2**: Forrest plot depicting the subgroup comparison of the effect of intensive blood pressure control on the incidence of MACE in elderly versus non‐elderly patients.
**Figure S3**: Forrest plot depicting the subgroup comparison of the effect of intensive blood pressure control on the incidence of MACE in patients with diabetes versus those without diabetes.
**Figure S4**: Forrest plot depicting the subgroup comparison of the effect of intensive blood pressure control on the incidence of MACE in patients with prior cardiovascular disease versus those without prior cardiovascular disease.
**Figure S5**: Forrest plot depicting the subgroup comparison of the effect of intensive blood pressure control on the incidence of MACE in trials with a mean baseline systolic blood pressure <145 versus >145 mm Hg.

## Data Availability

Data will be shared upon reasonable request.

## References

[1] Mills KT , Stefanescu A , He J . The global epidemiology of hypertension. Nat Rev Nephrol. 2020;16(4):223–237. 32024986 10.1038/s41581-019-0244-2 PMC7998524

[2] Dai H , Bragazzi NL , Younis A , Zhong W , Liu X , Wu J , et al. Worldwide trends in prevalence, mortality, and disability‐adjusted life years for hypertensive heart disease from 1990 to 2017. Hypertension. 2021;77(4):1223–1233. 33583201 10.1161/HYPERTENSIONAHA.120.16483

[3] Blood Pressure Lowering Treatment Trialists’ Collaboration . Pharmacological blood pressure lowering for primary and secondary prevention of cardiovascular disease across different levels of blood pressure: an individual participant‐level data meta‐analysis. Lancet. 2021;397(10285):1625–1636. 33933205 10.1016/S0140-6736(21)00590-0 PMC8102467

[4] Kreutz R , Brunström M , Burnier M , Grassi G , Januszewicz A , Muiesan ML , et al. 2024 European Society of Hypertension clinical practice guidelines for the management of arterial hypertension. Eur J Intern Med. 2024;126:1–15. 38914505 10.1016/j.ejim.2024.05.033

[5] Whelton PK , Carey RM , Aronow WS , Casey DE , Collins KJ , Dennison Himmelfarb C , et al. 2017 ACC/AHA/AAPA/ABC/ACPM/AGS/APhA/ASH/ASPC/NMA/PCNA guideline for the prevention, detection, evaluation, and management of high blood pressure in adults: a report of the American College of Cardiology/American Heart Association Task Force on Clinical Practice Guidelines. Hypertension. 2018;71(6):e13–e115. 29133356 10.1161/HYP.0000000000000065

[6] Bundy JD , Li C , Stuchlik P , Bu X , Kelly TN , Mills KT , et al. Systolic blood pressure reduction and risk of cardiovascular disease and mortality: a systematic review and network meta‐analysis. JAMA Cardiol. 2017;2(7):775–781. 28564682 10.1001/jamacardio.2017.1421 PMC5710614

[7] SPRINT Research Group , Lewis CE , Fine LJ , Beddhu S , Cheung AK , Cushman WC , et al. Final report of a trial of intensive versus standard blood‐pressure control. N Engl J Med. 2021;384(20):1921–1930. 34010531 10.1056/NEJMoa1901281 PMC9907774

[8] ACCORD Study Group , Cushman WC , Evans GW , Byington RP , Goff DC , Grimm RH , et al. Effects of intensive blood‐pressure control in type 2 diabetes mellitus. N Engl J Med. 2010;362(17):1575–1585. 20228401 10.1056/NEJMoa1001286 PMC4123215

[9] Mori Y , Mizuno A , Fukuma S . Low on‐treatment blood pressure and cardiovascular events in patients without elevated risk: a nationwide cohort study. Hypertens Res. 2024;47(6):1546–1554. 38355817 10.1038/s41440-024-01593-y PMC11150151

[10] Cumpston M , Li T , Page MJ , Chandler J , Welch VA , Higgins JP , et al. Updated guidance for trusted systematic reviews: a new edition of the Cochrane Handbook for Systematic Reviews of Interventions. Cochrane Database Syst Rev. 2019;10(10):ED000142. 31643080 10.1002/14651858.ED000142 PMC10284251

[11] Page MJ , McKenzie JE , Bossuyt PM , Boutron I , Hoffmann TC , Mulrow CD , et al. The PRISMA 2020 statement: an updated guideline for reporting systematic reviews. Syst Rev. 2021;10(1):89. 33781348 10.1186/s13643-021-01626-4 PMC8008539

[12] Liu J , Li Y , Ge J , Yan X , Zhang H , Zheng X , et al. Lowering systolic blood pressure to less than 120 mm Hg versus less than 140 mm Hg in patients with high cardiovascular risk with and without diabetes or previous stroke: an open‐label, blinded‐outcome, randomised trial. Lancet. 2024;404(10449):245–255. 38945140 10.1016/S0140-6736(24)01028-6

[13] Guyatt GH , Oxman AD , Vist GE , Kunz R , Falck‐Ytter Y , Alonso‐Coello P , et al. GRADE: an emerging consensus on rating quality of evidence and strength of recommendations. BMJ. 2008;336(7650):924–926. 18436948 10.1136/bmj.39489.470347.AD PMC2335261

[14] Bergmann F , Pracher L , Sawodny R , Blaschke A , Gelbenegger G , Radtke C , et al. Efficacy and safety of corticosteroid therapy for community‐acquired pneumonia: a meta‐analysis and meta‐regression of randomized, controlled trials. Clin Infect Dis. 2023;77(12):1704–1713. 37876267 10.1093/cid/ciad496

[15] Kitagawa K , Yamamoto Y , Arima H , Maeda T , Sunami N , Kanzawa T , et al. Effect of standard vs intensive blood pressure control on the risk of recurrent stroke: a randomized clinical trial and meta‐analysis. JAMA Neurol. 2019;76(11):1309–1318. 31355878 10.1001/jamaneurol.2019.2167 PMC6664380

[16] Bi Y , Li M , Liu Y , Li T , Lu J , Duan P , et al. Intensive blood‐pressure control in patients with type 2 diabetes. N Engl J Med. 2024 Nov 16. 10.1056/NEJMoa2412006 39555827

[17] Oparil S , Acelajado MC , Bakris GL , Berlowitz DR , Cífková R , Dominiczak AF , et al. Hypertension. Nat Rev Dis Primers. 2018;4:18014. 29565029 10.1038/nrdp.2018.14 PMC6477925

[18] Weiss J , Freeman M , Low A , Fu R , Kerfoot A , Paynter R , et al. Benefits and harms of intensive blood pressure treatment in adults aged 60 years or older: a systematic review and meta‐analysis. Ann Intern Med. 2017;166(6):419–429. 28114673 10.7326/M16-1754

[19] McEvoy JW , McCarthy CP , Bruno RM , Brouwers S , Canavan MD , Ceconi C , et al. 2024 ESC Guidelines for the management of elevated blood pressure and hypertension. Eur Heart J. 2024;45(38):3912–4018. 39210715 10.1093/eurheartj/ehae178

[20] Nazarzadeh M , Bidel Z , Canoy D , Copland E , Bennett DA , Dehghan A , et al. Blood pressure‐lowering treatment for prevention of major cardiovascular diseases in people with and without type 2 diabetes: an individual participant‐level data meta‐analysis. Lancet Diabetes Endocrinol. 2022;10(9):645–654. 35878651 10.1016/S2213-8587(22)00172-3 PMC9622419

[21] Brunström M , Carlberg B . Effect of antihypertensive treatment at different blood pressure levels in patients with diabetes mellitus: systematic review and meta‐analyses. BMJ. 2016;352:i717. 26920333 10.1136/bmj.i717 PMC4770818

[22] Wright JT , Whelton PK , Johnson KC , Snyder JK , Reboussin DM , Cushman WC , et al. SPRINT revisited: updated results and implications. Hypertension. 2021;78(6):1701–1710. 34757768 10.1161/HYPERTENSIONAHA.121.17682 PMC8824314

[23] Kjeldsen SE , Mancia G . Unattended automated office vs. ambulatory blood pressure in people with high cardiovascular risk: implications for understanding the SPRINT results. J Hypertens. 2019;37(1):6–8. 30499916 10.1097/HJH.0000000000001874

[24] Seo J , Lee CJ , Oh J , Lee SH , Kang SM , Park S . Large discrepancy between unobserved automated office blood pressure and ambulatory blood pressure in a high cardiovascular risk cohort. J Hypertens. 2019;37(1):42–49. 30507862 10.1097/HJH.0000000000001868

[25] SPS3 Study Group , Benavente OR , Coffey CS , Conwit R , Hart RG , McClure LA , et al. Blood‐pressure targets in patients with recent lacunar stroke: the SPS3 randomised trial. Lancet. 2013;382(9891):507–515. 23726159 10.1016/S0140-6736(13)60852-1 PMC3979302

[26] Mant J , McManus RJ , Roalfe A , Fletcher K , Taylor CJ , Martin U , et al. Different systolic blood pressure targets for people with history of stroke or transient ischaemic attack: PAST‐BP (prevention after stroke–blood pressure) randomised controlled trial. BMJ. 2016;352:i708. 26919870 10.1136/bmj.i708 PMC4770816

[27] Bath PM , Scutt P , Blackburn DJ , Ankolekar S , Krishnan K , Ballard C , et al. Intensive versus guideline blood pressure and lipid lowering in patients with previous stroke: main results from the pilot ‘prevention of decline in cognition after stroke trial’ (podcast) randomised controlled trial. PLoS ONE. 2017;12(1):e0164608. 28095412 10.1371/journal.pone.0164608 PMC5240987

[28] Beddhu S , Greene T , Boucher R , Cushman WC , Wei G , Stoddard G , et al. Intensive systolic blood pressure control and incident chronic kidney disease in people with and without diabetes mellitus: secondary analyses of two randomised controlled trials. Lancet Diabetes Endocrinol. 2018;6(7):555–563. 29685860 10.1016/S2213-8587(18)30099-8 PMC6071316

[29] Beddhu S , Rocco MV , Toto R , Craven TE , Greene T , Bhatt U , et al. Effects of intensive systolic blood pressure control on kidney and cardiovascular outcomes in persons without kidney disease: a secondary analysis of a randomized trial. Ann Intern Med. 2017;167(6):375–383. 28869987 10.7326/M16-2966 PMC8545525

[30] PROGRESS Collaborative Group . Randomised trial of a perindopril‐based blood‐pressure‐lowering regimen among 6,105 individuals with previous stroke or transient ischaemic attack. Lancet. 2001;358(9287):1033–1041. 11589932 10.1016/S0140-6736(01)06178-5

[31] Heart Outcomes Prevention Evaluation Study Investigators , Yusuf S , Sleight P , Pogue J , Bosch J , Davies R , et al. Effects of an angiotensin‐converting‐enzyme inhibitor, ramipril, on cardiovascular events in high‐risk patients. N Engl J Med. 2000;342(3):145–153. 10639539 10.1056/NEJM200001203420301

[32] Jamerson K , Weber MA , Bakris GL , Dahlöf B , Pitt B , Shi V , et al. Benazepril plus amlodipine or hydrochlorothiazide for hypertension in high‐risk patients. N Engl J Med. 2008;359(23):2417–2428. 19052124 10.1056/NEJMoa0806182

[33] Jorda A , Zeitlinger M , Jilma B , Gelbenegger G . Patient‐level and trial‐level data meta‐analyses. Lancet. 2024;404(10449):242–243. 39033006 10.1016/S0140-6736(24)00863-8

[34] Wang W , Jiang B , Sun H , Ru X , Sun D , Wang L , et al. Prevalence, incidence, and mortality of stroke in china: results from a nationwide population‐based survey of 480 687 adults. Circulation. 2017;135(8):759–71. 28052979 10.1161/CIRCULATIONAHA.116.025250

