# Supplementary material for: Systolic blood pressure targets below 120 mm Hg are associated with reduced mortality: A meta‐analysis
Source: J Intern Med. 2025 Mar 5;297(5):479–91. doi: 10.1111/joim.20078 (PMC12032999; doi:10.1111/joim.20078)
Supplement: Supplementary file 1 — Table S1: Precise search strategy and number of records found in each database (Search conducted on November 28, 2024). Table S2: Definition of the outcome MACE of included studies. Table S3: Definition of elderly versus non‐elderly for subgroup analysis. Table S4: Extended trial characteristics. Table S5: Baseline characteristics of the study populations in the included trials. Table S6: Assessment of level of certainty of evidence according to GRADE recommendations. Table S7: Risk of Bias Assessment according to the Revised Cochrane risk‐of‐bias tool for randomized trials (RoB 2). Figure S1: Forrest plot depicting the subgroup comparison of the effect of intensive blood pressure control on the incidence of MACE in male versus female patients. Figure S2: Forrest plot depicting the subgroup comparison of the effect of intensive blood pressure control on the incidence of MACE in elderly versus non‐elderly patients. Figure S3: Forrest plot depicting the subgroup comparison of the effect of intensive blood pressure control on the incidence of MACE in patients with diabetes versus those without diabetes. Figure S4: Forrest plot depicting the subgroup comparison of the effect of intensive blood pressure control on the incidence of MACE in patients with prior cardiovascular disease versus those without prior cardiovascular disease. Figure S5: Forrest plot depicting the subgroup comparison of the effect of intensive blood pressure control on the incidence of MACE in trials with a mean baseline systolic blood pressure <145 versus >145 mm Hg. [file JOIM-297-479-s001.docx]

**Supplementary Appendix**

**Intensive versus standard BP control**

Felix Bergmann^1^, Marlene Prager^1^, Lena Pracher^1^, Rebecca Sawodny^2^, Gloria M. Steiner-Gager^1^, Bernhard Richter^3^, Bernd Jilma^1^, Markus Zeitlinger^1^, Georg Gelbenegger^1^, Anselm Jorda^1^

^1^Department of Clinical Pharmacology, Medical University of Vienna, Vienna, Austria

^2^Department of Pathology, Medical University of Vienna, Vienna, Austria

^3^Department of Medicine II, Division of Cardiology, Medical University of Vienna, Vienna, Austria

Supplementary Table S 1 Precise search strategy and number of records found in each database (Search conducted on November 28, 2024).

| **Pubmed**  ((blood pressure[Title/Abstract]) OR (hypertension[Title/Abstract])) AND ((cardiovascular risk[Title/Abstract]) OR (myocardial infarction[Title/Abstract]) OR (stroke[Title/Abstract]) OR (heart failure[Title/Abstract])) AND ((120 mm Hg[Title/Abstract]) OR (intensive blood pressure[Title/Abstract])) | **N=**  **667** |
| --- | --- |
| (((blood pressure[Title/Abstract]) OR (hypertension[Title/Abstract])) AND ((cardiovascular risk[Title/Abstract]) OR (myocardial infarction[Title/Abstract]) OR (stroke[Title/Abstract]) OR (heart failure[Title/Abstract])) AND ((120 mm Hg[Title/Abstract]) OR (intensive blood pressure[Title/Abstract])) ) AND (randomized[Title/Abstract] OR randomised[Title/Abstract] OR randomly[Title/Abstract]) | **280** |
| **Embase**  ('blood pressure':ab,ti OR hypertension:ab,ti) AND ('cardiovascular risk':ab,ti OR 'myocardial infarction':ab,ti OR stroke:ab,ti OR 'heart failure':ab,ti) AND ('120 mm hg':ab,ti OR 'intensive blood pressure':ab,ti) | **929** |
| ('blood pressure':ab,ti OR hypertension:ab,ti) AND ('cardiovascular risk':ab,ti OR 'myocardial infarction':ab,ti OR stroke:ab,ti OR 'heart failure':ab,ti) AND ('120 mm hg':ab,ti OR 'intensive blood pressure':ab,ti) AND (randomized:ab,ti OR randomised:ab,ti OR randomly:ab,ti) | **413** |
| **Web of Science**  ((TI=(blood pressure) OR TI=(hypertension)) AND (TI=(cardiovascular risk) OR TI=(myocardial infarction) OR TI=(stroke) OR TI=(heart failure)) AND (TI=(120 mmHg) OR TI=(intensive blood pressure))) OR ((AB=(blood pressure) OR AB=(hypertension)) AND (AB=(cardiovascular risk) OR AB=(myocardial infarction) OR AB=(stroke) OR AB=(heart failure)) AND (AB=(120 mmHg) OR AB=(intensive blood pressure))) | **3297** |
| ((TI=(blood pressure) OR TI=(hypertension)) AND (TI=(cardiovascular risk) OR TI=(myocardial infarction) OR TI=(stroke) OR TI=(heart failure)) AND (TI=(120 mmHg) OR TI=(intensive blood pressure))) OR ((AB=(blood pressure) OR AB=(hypertension)) AND (AB=(cardiovascular risk) OR AB=(myocardial infarction) OR AB=(stroke) OR AB=(heart failure)) AND (AB=(120 mmHg) OR AB=(intensive blood pressure))) AND (TI=(randomized) OR AB=(randomized) OR TI=(randomised) OR AB=(randomised) OR TI=(randomly) OR AB=(randomly)) | **973** |
| **Cochrane Library**  (blood pressure OR hypertension):ti,ab,kw AND (cardiovascular risk OR myocardical infarction OR stroke OR heart failure):ti,ab,kw AND (120 mm Hg OR intensive blood pressure):ti,ab,kw | **4407** |
| (blood pressure OR hypertension):ti,ab,kw AND (cardiovascular risk OR myocardical infarction OR stroke OR heart failure):ti,ab,kw AND (120 mm Hg OR intensive blood pressure):ti,ab,kw AND (randomized OR randomised OR randomly):ti,ab,kw | **3574** |

Supplementary Table S 2 Definition of the outcome MACE of included studies

| **Reference** | **Year** | **Definition of MACE** |
| --- | --- | --- |
| ACCORD | 2010 | Nonfatal myocardial infarction, nonfatal stroke or cardiovascular death |
| RESPECT | 2019 | Cardiovascular death, nonfatal stroke, and nonfatal myocardial infarction |
| SPRINT | 2021 | Myocardial infarction, acute coronary syndrome not resulting in myocardial infarction, stroke, acute decompensated heart failure, or death from cardiovascular causes |
| ESPRIT | 2024 | Myocardial infarction, acute coronary syndrome not resulting in myocardial infarction, stroke, acute decompensated heart failure, or death from cardiovascular causes |
| BPROAD | 2024 | Nonfatal stroke, nonfatal myocardial infarction, treatment or hospitalization for heart failure, or death from cardiovascular causes |

Supplementary Table S 3 Definition of elderly versus non-elderly for subgroup analysis

| **Reference** | **Year** | **Non-Elderly**  **(age in years)** | **Elderly**  **(age in years)** |
| --- | --- | --- | --- |
| ACCORD | 2010 | <65 | ≥65 |
| SPRINT | 2021 | <75 | ≥75 |
| ESPRIT | 2024 | <70 | ≥70 |
| BPROAD | 2024 | <65 | ≥65 |

Supplementary Table S 4 Extended trial characteristics

| **Reference** | **Year** | **Recruitment Dates** | **Blinding** | **Randomized** | **Sample Size Total** | **Sample Size Intensive Therapy** | **Sample Size Standard Therapy** | **Medication used** | **Primary Outcomes** | **Secondary Outcomes** | **Main Safety Outcomes** | **Key Inclusion Criteria** | **Key Exclusion Criteria** | **Mortality** | **Measurement Method** |
| --- | --- | --- | --- | --- | --- | --- | --- | --- | --- | --- | --- | --- | --- | --- | --- |
| ACCORD | 2010 | 2001-2005 | non-blinded | yes | 4733 | 2362 | 2371 | ACE inhibitors  Diuretics  β-blockers  dihydro-pyridine and nondihydropyridine CCBs  α-blockers  angiotensin II receptor blockers sympatholytics  α-/β- blockers  thiazide diuretic + potassium-sparing diuretic  β-blocker + diuretic  ACE inhibitor + diuretic  ARB + diuretic  dihydropyridine CCB + ACE inhibitor | MACE | Expanded macrovascular outcome (MACE + revascularization or hospitalization for congestive heart failure) Major coronary disease event (fatal coronary event, nonfatal myocardial infarction or unstable angina) Nonfatal myocardial infarction Fatal stroke Nonfatal stroke All-cause mortality Death from cardiovascular causes Hospitalization due to heart failure Death due to heart failure | Hypotension Syncope Bradycardia or arrhythmia Hyperkalemia Angioedema Renal failure Hives or Swelling Dizziness when standing Potassium <3.2 or >5.9 mmol/L Elevation in serum creatine GFR < 30 mL/min/1.73m^3 | Type 2 diabetes mellitus Glycated hemoglobin level of 7.5% or more  ≥40 years of age + cardiovascular or ≥55 years of age + anatomical evidence of a substantial amount of atherosclerosis, albuminuria, left ventricular hy- pertrophy, or at least two additional risk factors for cardiovascular disease (dyslipidemia, hypertension, smoking, or obesity). | Body-mass index >45 Serum creatinine level >1.5 mg/dL Other serious illnesses | Mean mortality follow up: 5 years | Mean of three seated measurements after 5 minutes rest |
| ESPRIT | 2024 | 2019-2020 | blinded-outcome | yes | 11255 | 5624 | 5631 | angiotensin converting enzyme inhibitors  angiotensin receptor blockers  calcium channel blockers  thiazide type diuretics  β blockers | MACE | Myocardial infarction Coronary or non-coronary revascularisation Hospitalization or emergency room visit for heart failure Stroke Death from cardiovascular causes All-cause mortality Composite kidney outcome (ie, end-stage renal disease, a sustained decline in eGFR to <10 mL/min per 1∙73 m2, death from renal causes, or a sustained decline ≥40% in eGFR from baseline). | Any SAE Hypotension Syncope Electrolyte abnormality Injurious fall Acute kidney injury | Aged ≥50 years, systolic blood pressure betweeen 130-180 mm Hg, high cardiovascular risk (ie, established cardiovascular disease or at least two major cardiovascular risk factors such as aged 60 years or older for men or 65 years or older for women, diabetes, dyslipidemia, current smoker) | Secondary cause of hypertension, one-minute standing systolic blood pressure of less than 110 mm Hg, scheduled revascularisation within the next 6 months, left ventricular ejection fraction of less than 35%, estimated glomerular filtration rate (eGFR) of less than 45 mL/min per 1∙73 m² | Not stated | Mean of three seated measurements after 5 minutes rest |
| RESPECT | 2019 | 2010-2017 | masked-endpoint | yes | 1263 | 633 | 630 | Combination drug of losartan potassium or other angiotensin II receptor blockers  hydrochlorothiazide amlodipine besylate  spironolactone | Recurrent stroke, including ischemic stroke and intracerebral hemorrhage | Reductions in ischemic stroke Subtype of ischemic stroke (including atherothrombotic infarction, cardioembolic infarction, lacunar infarction, or infarction due to other and un- known etiology) Intracerebral hemorrhage Subarachnoid hemorrhage Transient ischemic attack Myocardial infarction MACE (cardiovascular death, nonfatal stroke, and nonfatal myocardial infarction) All cause mortality Composite of all-cause death, nonfatal stroke, and nonfatal myocardial infarction. | SAE monitoring (Includes: Angina pectoris New-onset or worsening heart failure New-onset or worsening of atrial fibrillation Coronary intervention or surgery Aortic aneurysm rupture /dissection New-onset or worsening of peripheral artery disease Worsening renal function* Hemodialysis treatment New-onset diabetes mellitus New-onset gout New-onset or worsening cognitive function Abnormality of serum potassium Malignant neoplasm Bone fracture Pneumonia Syncope / dizziness Infectious disease Collagen or blood disease Endocrine or metabolic disease Psychiatric or neurologic disease Opthalmic or skin disease Acute or chronic subdural hematoma Other cardiovascular disease Respiratory disease Gastrointestinal disease Musculoskeletal disorders Kidney / urinary tract Genital system disease Injury Dehydration, abnormal sodium and others) | Age 50 to 85 years, independent ambulation, systolic BP of 130 to 180mmHg or diastolic BP of 80 to 110 mmHg on a regimen of 0 to 3 antihypertensive medications, and history of stroke within the previous 3 years (evidence of an acute disturbance of focal neurological functions, with symptoms lasting more than 24 hours, and symptomatic ischemic stroke or intracerebral hemorrhage confirmed by magnetic resonance imaging or computed tomography) | Patients older than 85 years, stroke onset occurred 1 month or less previously | Not stated | Not stated |
| SPRINT | 2021 | 2010-2013 | masked-endpoint | yes | 9361 | 4678 | 4683 | ACE-Inhibitors  angiotensin-receptor blockers diuretics  calcium channel blockers  β blockers  vasodilators  alpha 2 agonists  alpha blockers | MACE | Myocardial infarction Acute coronary syndrome (not resulting in myocardial infarction) Stroke Acute decompensated heart failure Death from cardiovascular cause All cause mortality MACE + All cause mortality | Any SAE Hypotension Syncope Bradycardia Electrolyte abnormality Injurious fall Acute kidney injury or acute renal failure | Aged ≥50 years, systolic blood pressure 130-180 mm Hg, at least one additional indicator of cardiovascular risk (clinical or subclinical cardiovascular disease, chronic kidney disease (defined as an estimated glomerular filtration rate [eGFR] of 20 to 59 ml per minute per 1.73 m2 of body-surface area), a 15% or greater 10-year risk of cardiovascular disease as determined on the basis of the Framingham cardiovascular risk score, or an age of 75 years or older) | Diabetes mellitus, previous stroke, dementia, known secondary cause of hypertension, one-minute standing SBP < 110 mm Hg, eGFR < 20 ml/min /1.73m2 or end-stage renal disease, Cardiovascular event or procedure or hospitalization for unstable angina within last 3 months, symptomatic heart failure within the past 6 months or left ventricular ejection fraction < 35% | Median mortality follow up: 3.33 years during the intervention period, extended to 3.88 years including post-trial follow-up | Mean of three seated measurements after 5 minutes rest |
| BPROAD | 2024 | 2019-2021 | blinded- outcome | yes | 12821 | 6414 | 6407 | ACE-Inhibitors, Angiotensin-receptor blockers, calcium channel blockers, diuretics, α-/β- receptor blockers | MACE | Fatal or nonfatal stroke, fatal or nonfatal myocardial infarction,  treatment or hospitalization for heart failure, death from cardiovascular causes, death from any cause, expanded composite of the primary outcome or death from any cause, chronic kidney disease (CKD) outcomes (progression of CKD, development of CKD, incident albuminuria) | Any SAEs, arrhythmias, electrolyte abnormalities, injurious falls, symptomatic hypotension, syncope, acute renal failure, abnormal serum sodium or potassium concentrations | Type 2 diabetes,  aged ≥50 years, elevated  systolic blood pressure (130 to 180 mmHg in patients taking antihypertensive  medications or at least 140 mmHg in patients not taking medications), and increased risk of cardiovascular disease (one or more of the following criteria: a history of clinical cardiovascular disease at least 3 months before enrolment in the trial, subclinical cardiovascular  disease within 3 years before enrolment,  two or more cardiovascular disease risk factors, and chronic kidney disease (CKD)) | Type 1 diabetes, secondary cause of hypertension, low standing systolic BP (<110 mmHg), recent cardiovascular event/procedure or hospitalization for unstable angina (<3 months), symptomatic heart failure or LVEF <35% (<6 months), elevated liver enzymes, severe renal dysfunction (dialysis, eGFR <30, serum creatinine >2.0 mg/dL), significant proteinuria, polycystic kidney disease/glomerulonephritis | Median mortality follow-up: 4.2 years | Mean of three seated measurements after 5 minutes rest |

Supplementary Table S 5 Baseline characteristics of the study populations in the included trials.

|  | **ACCORD 2010** | | **BPROAD 2024** | | **ESPRIT 2024** | | **RESPECT 2019** | | **SPRINT 2021** | |
| --- | --- | --- | --- | --- | --- | --- | --- | --- | --- | --- |
|  | Intensive Group | Control Group | Intensive Group | Control Group | Intensive Group | Control Group | Intensive Group | Control Group | Intensive Group | Control Group |
| Age, mean ± SD | 62.2 ± 6.8 | 62.2 ± 6.9 | 63.7 ± 7.4 | 63.9 ± 7.5 | 64.6 ± 7.1 | 64.6 ± 7.2 | 67.2 ± 8.8 | 67.3 ± 8.8 | 67.9 ± 9.4 | 67.9 ± 9.5 |
| Female sex, no. (%) | 1128 (47.8) | 1130 (47.7) | 2923 (45.6) | 2880 (45.0) | 2327 (41.4) | 2323 (41.3) | 184 (29.1) | 202 (32.1) | 1684 (36.0) | 1648 (35.2) |
| BMI, mean ± SD | 32.2 ± 5.7 | 32.1 ± 5.4 | 26.7 ± 3.2 | 26.7 ± 3.3 | 26.3 ± 3.3 | 26.3 ± 3.3 | 23.7 ± 3.2 | 23.9 ± 3.3 | 29.9 ± 5.8 | 29.8 ± 5.7 |
| Diabetes, no. (%) | 2362 (100) | 2371 (100) | 6414 (100) | 6407 (100) | 2180 (38.8) | 2179 (38.7) | 142 (22.4) | 154 (24.4) | 0 (0) | 0 (0) |
| History of dyslipidemia, no. (%) | N.A. | N.A. | N.A. | N.A. | N.A. | N.A. | 224 (35.4) | 234 (37.1) | N.A. | N.A. |
| History of coronary heart disease, no. (%) | N.A. | N.A. | N.A. | N.A. | 1632 (29.0) | 1620 (28.8) | 15 (2.4) | 17 (2.7) | N.A. | N.A. |
| Previous cardiovascular event, no. (%) | 804 (34.0) | 789 (33.3) | 1480 (23.1) | 1408 (22.0) | N.A. | N.A. | N.A. | N.A. | 940 (20.1) | 937 (20.0) |
| Previous heart failure, no. (%) | 109 (4.7) | 94 (4.0) | N.A. | N.A. | N.A. | N.A. | N.A. | N.A. | N.A. | N.A. |
| Previous stroke, no. (%) | N.A. | N.A. | N.A. | N.A. | 1520 (27.0) | 1502 (26.7) | 633 (100) | 630 (100) | 0 | 0 |
| Previous peripheral artery disease, no. (%) | N.A. | N.A. | N.A. | N.A. | 44 (0.8) | 28 (0.5) | N.A. | N.A. | N.A. | N.A. |
| Previous abdominal aortic aneurysm, no. (%) | N.A. | N.A. | N.A. | N.A. | 3 (0.1) | 4 (0.1) | N.A. | N.A. | N.A. | N.A. |
| Previous atrial fibrillation, no. (%) | N.A. | N.A. | N.A. | N.A. | 113 (2.0) | 112 (2.0) | 53 (8.4) | 53 (8.4) | N.A. | N.A. |
| Chronic kidney disease, no. (%) | N.A. | N.A. | 501 (7.8) | 469 (7.3) | N.A. | N.A. | 36 (5.7) | 27 (4.3) | 1329 (28.4) | 1316 (28.1) |
| Current smoker, no. (%) | 314 (13.3) | 312 (13.2) | 1580/6401 (24.7) | 1636/6392 (25.6) | 1739 (30.9) | 1777 (31.6) | N.A. | N.A. | 639 (13.7) | 601 (12.8) |
| Baseline systolic BP, mean ± SD | 139 ± 16.1 | 139.4 ± 15.5 | 140.0 ± 10.2 | 140.4 ± 10.2 | 146.8 ± 10.5 | 147.0 ± 10.7 | 145.1 ± 12.4 | 145.7 ± 12.9 | 139.7 ± 15.8 | 139.7 ± 15.4 |
| Baseline diastolic BP, mean ± SD | 75.9 ± 10.6 | 76.0 ± 10.2 | 76.3 ± 9.2 | 76.3 ± 9.1 | 82.8 ± 10.1 | 82.9 ± 10.5 | 83.6 ± 10.7 | 83.7 ± 10.4 | 78.2 ± 11.9 | 78.0 ± 12.0 |
| Achieved systolic BP, mean ± SD or median (IQR) | 119.3 | 133.8 | 121.6 | 133.2 | 119.1 ± 11.1 | 134.8 ± 10.5 | 126.7 (125.9-127.2) | 133.2 (132.5-133.8) | 120.0 | 133.9 |
| Achieved diastolic BP, mean ± SD or median (IQR) | 64.4 | 70.5 | N.A. | N.A. | N.A. | N.A. | 74.4 (73.8-75.0) | 77.7 (77.1-78.4) | N.A. | N.A. |
| Cholesterol (mg/L), mean ± SD |  |  |  |  |  |  |  |  |  |  |
| Total | 194.1 ± 45.1 | 191.4 ± 44.3 | 157.8 ± 48.1 | 157.2 ± 47.8 | 4.0 ± 1.2 (mmol/L) | 4.0 ± 1.2 (mmol/L) | N.A. | N.A. | 190.2 ± 41.4 | 190.0 ± 40.9 |
| Low-density lipoprotein | 111.1 ± 37.4 | 108.8 ± 36 | 84.3 ± 34.6 | 83.7 ± 34.1 | 2.3 ± 0.8 (mmol/L) | 2.3 ± 0.8 (mmol/L) | 111.8 ± 32.2 | 112.0 ± 34.5 | N.A. | N.A. |
| High-density lipoprotein | 46.5 ± 13.4 | 46.9 ± 14.2 | 40.2 ± 12.7 | 40.1 ± 11.8 | 0.9 ± 0.3 (mmol/L) | 0.9 ± 0.3 (mmol/L) | 54.4 ± 15.5 | 54.1 ± 14.9 | 52.9 ± 14.3 | 52.8 ± 14.6 |
| Total plasma triglycerides (mg/L), mean ± SD or median (IQR) | 147 (98-227) | 147 (98-224) | 130.2 (92.1–192.2) | 131.1 (92.1–194.0) | 1.7 ± 1.1 (mmol/L) | 1.7 ± 1.1 (mmol/L) | N.A. | N.A. | 124.8 ± 85.8 | 127.1 ± 95.0 |
| Estimated GFR (ml/min/1.73m^2), mean ± SD | 91.6 ± 30.3 | 91.7 ± 27.1 | 88.6 ± 17.8 | 88.7 ± 18.0 | 83.2 ± 13.6 | 83.5 ± 13.7 | N.A. | N.A. | 71.8 ± 20.7 | 71.7 ± 20.5 |
| Antihypertensive drugs at entry - no. of medications, mean | N.A. | N.A. | 1.4 ± 0.6 | 1.4 ± 0.6 | N.A. | N.A. | 1.5 | 1.4 | 1.8 (1.0) | 1.8 (1.0) |
| Concomitant drugs at entry, no. (%) - Statin | 1509 (63.9) | 1555 (65.6) | 4192 (65.4) | 4159 (64.9) | 2623 (46.6) | 2591 (46.0) | 210 (33.2) | 225 (35.7) | 1978 (42.6) | 2076 (44.7) |
| Concomitant drugs at entry, no. (%) - Antiplatelet drug | 1323 (56.0) | 1252 (52.8) | 3225 (50.3) | 3200 (49.9) | 2419 (43.0) | 2398 (42.6) | 442 (69.8) | 455 (72.2) | 2406 (51.6) | 2350 (50.4) |
| Concomitant drugs at entry, no. (%) - Anticoagulant drug | N.A. | N.A. | N.A. | N.A. | N.A. | N.A. | 68 (10.7) | 68 (10.8) | N.A. | N.A. |

Supplementary Table S 6 Assessment of level of certainty of evidence according to GRADE recommendations

| **Outcome** | | **All-cause mortality at 1 years** |
| --- | --- | --- |
| Trials | | 5 RCTs  all open label |
| Number of patients (intention-to-treat population) | | 39,434  (19,712 vs. 19,722) |
| Pooled effect (95% CI) | | Risk Ratio 0.87  95% CI 0.76 to 0.99  P = 0.03 |
| Down-grading  factors | Risk of bias | Some |
|  | Imprecision | Low |
|  | Inconsistency | Low |
|  | Indirectness | Low |
|  | Publication bias | Low |
| Up-grading factors | Large magnitude of effect | No |
|  | Dose-response gradient | n.a. |
|  | All residual confounding would decrease magnitude of effect | n.a. |
| **Level of certainty** | | Low |

Supplementary Table S 7 Risk of Bias Assessment according to the Revised Cochrane risk-of-bias tool for randomized trials (RoB 2)

| **Study** | Domain 1: Risk of bias arising from the randomization process | Domain 2: Risk of bias due to deviations from the intended interventions (*effect of assignment to intervention*) | Domain 2: Risk of bias due to deviations from the intended interventions (*effect of adhering to intervention*) | Domain 3: Risk of bias due to missing outcome data | Domain 4: Risk of bias in measurement of the outcome | Domain 5: Risk of bias in selection of the reported result | **Overall risk of bias** |
| --- | --- | --- | --- | --- | --- | --- | --- |
| **ACCORD 2010** | Low | Some | Some | Low | Low | Low | **Some risk** |
| **ESPRIT 2024** | Low | Some | Some | Low | Low | Low | **Some risk** |
| **BPROAD 2024** | Low | Some | Some | Low | Low | Low | **Some risk** |
| **RESPECT 2019** | Low | Some | Some | Low | Low | Low | **Some risk** |
| **SPRINT 2021** | Low | Some | Some | Low | Low | Low | **Some risk** |

Supplementary Figure S 1 Forrest plot depicting the subgroup comparison of the effect of intensive blood pressure control on the incidence of MACE in male versus female patients

Supplementary Figure S 2 Forrest plot depicting the subgroup comparison of the effect of intensive blood pressure control on the incidence of MACE in elderly versus non-elderly patients

Supplementary Figure S 3 Forrest plot depicting the subgroup comparison of the effect of intensive blood pressure control on the incidence of MACE in patients with diabetes versus those without diabetes

Supplementary Figure S 4 Forrest plot depicting the subgroup comparison of the effect of intensive blood pressure control on the incidence of MACE in patients with prior cardiovascular disease versus those without prior cardiovascular disease

Supplementary Figure S 5 Forrest plot depicting the subgroup comparison of the effect of intensive blood pressure control on the incidence of MACE in trials with a mean baseline systolic blood pressure <145 versus >145 mm Hg.
